# Supplementary material for: Comparative analysis of the genomes of Stylophora pistillata and Acropora digitifera provides evidence for extensive differences between species of corals
Source: Sci Rep. 2017 Dec 14;7:17583. doi: 10.1038/s41598-017-17484-x (PMC5730576; doi:10.1038/s41598-017-17484-x)
Supplement: Supplementary file 1 — Supplementary Information [file 41598_2017_17484_MOESM1_ESM.pdf]

Supplementary Information

Comparative analysis of the genomes of *Stylophora pistillata* and *Acropora digitifera* provides evidence for extensive differences between species of corals

Christian R Voolstra[1,*], Yong Li[1], Yi Jin Liew[1], Sebastian Baumgarten[1+], Didier Zoccola[2], Jean-François Flot[3], Sylvie Tambutte[2], Denis Allemand[2], Manuel Aranda[1,*]

[1]Red Sea Research Center, Division of Biological and Environmental Science and Engineering (BESE), King Abdullah University of Science and Technology (KAUST), Thuwal, Saudi Arabia

[2]Centre Scientifique de Monaco, 8 quai Antoine Ier, Monaco, 98000, Monaco

[3]Université libre de Bruxelles, Avenue Franklin Roosevelt 50, 1050 Bruxelles, Belgium

[+]Current address: Biology of Host-Parasite Interactions Unit, Institut Pasteur, 25 rue du Dr Roux, 75015 Paris, France

*Corresponding Authors:

christian.voolstra@kaust.edu.sa

Tel: +966 12 8082377, Fax: +966 21 8082377

manuel.aranda@kaust.edu.sa

Tel: +966 12 8082979, Fax: +966 21 8082979

Short title: Extensive differences between coral genomes

Keywords: Coral Reef, Adaptive Evolution, Genome Sequencing, Scleractinia, Environmental Change, *Stylophora pistillata*, *Acropora digitifera*

Supplemental Tables

Table S1. Assembly statistics for the *Stylophora pistillata* genome assembly.

Overview statistics		
	Genome size (FACS)	434 Mbp
	Genome size (ALLPATHS-LG)	433 Mbp
	Total size of scaffolds	400,108,361 bp
	Number of scaffolds	5,688
	N50 scaffold length	457,453 bp
	Total size of contigs	358,078,850 bp
	Number of contigs	32,144
	N50 contig length	24,388 bp
	Average length of gaps (for gaps of >25 Ns)	1,589
Scaffold statistics		
	Longest scaffold	2,969,787 bp
	Shortest scaffold	891 bp
	Number of scaffolds >1 kb	5,565
	Number of scaffolds >10 kb	2,276
	Number of scaffolds >100 kb	857
	Number of scaffolds >1 Mb	59
	Mean scaffold size	70,343 bp
	Median scaffold size	4,680 bp
Contig statistics		
	Longest contig	252,210 bp
	Number of contigs >1 kb	31,587
	Number of contigs >10 kb	10,373
	Number of contigs >100 kb	104
	Mean contig size	11,140 bp
	Median contig size	5,088 bp

Table S2. Annotation statistics for the *Stylophora pistillata* gene models.

Total number of gene models	25,769	100%
**Total number of gene models with associated GO terms based on all UniProt annotations**	**21,446**	**83%**
Number of gene models with associated GO terms based on SwissProt annotations	17,506	68%
Number of gene models with associated GO terms based on TrEMBL annotations	3,940	15%
**Number of remaining gene models annotated against nr database**	**1,466**	**6%**
Number of gene models with KEGG annotations	12,623	49%
Number of gene models with PANTHER annotations	19,751	77%
Number of gene models with Pfam domains	17,951	70%

Table S3. No. of Core Eukaryotic Genes (CEGs) identified with CEGMA analysis in the genomes of the corals *Stylophora pistillata* (this study) and *Acropora digitifera* (Shinzato *et al.* 2011).

**No. of CEGs**	***Stylophora pistillata***	***Acropora digitifera***
248	235	204
%	94.76	82.26

Table S4. Sequencing libraries and NCBI accession numbers (PRJNA281535) used for *S. pistillata* genome assembly and gap filling. Library abbreviations are as follows: pe = paired-end, mp = mate-pair.

Library code	NCBI SRA accession no.	Insert size	No. of sequence reads before filtering	No. of sequences reads after filtering	Used for assembly	Used for Gap filling
pe06	SRX999654	190bp	136,879,629	122,574,751	+	+
pe07	SRX999933	190bp	134,613,054	122,295,744	+	+
pe18	SRX999934	200bp	151,771,736	142,887,569	+	+
pe19	SRX999935	200bp	54,003,303	51,957,308	+	+
miseq	SRX999949	400bp	14,970,181	14,133,302		+
mp01	SRX999936	3kb	34,282,529	18,555,836	+	+
mp02	SRX999939	5kb	21,433,929	16,296,876		+
mp03	SRX999941	8kb	18,535,761	12,906,205		+
mp04	SRX999942	3kb	88,066,688	64,606,537	+	+
mp05	SRX999943	3kb	207,104,853	195,072,501		+
mp06	SRX999944	6.2kb	38,432,387	20,922,528	+	+
mp07	SRX999945	7.8kb	38,172,153	19,473,493		+
mp08	SRX999947	10kb	36,477,254	18,813,296		+
mp09	SRX999948	11kb	44,567,883	23,523,074	+	+

Table S5. *Stylophora pistillata* genome repeat content.

Element [a]	Number of occurrences [b]	Number of bp covered [b]
**DNA Transposon**	**27,594**	**7,285,572**
MuDR	290	42,694
Tc2	1	105
EnSpm	2,235	502,539
Mirage	1	41
Mariner	3,396	522,041
Crypton	2,053	382,267
Rehavkus	2	83
Polinton	3,683	1,530,027
Sola	2,024	657,414
Tc1	2	101
Charlie	1	48
Helitron	1,121	322,541
ISL2EU	1,993	412,054
P	1,026	309,099
Merlin	827	160,993
hAT	2,239	453,296
Harbinger	2,358	626,912
Kolobok	782	311,592
Chapaev	62	18,491
PiggyBac	201	83,943
Novosib	576	20,020
IS4	2,716	928,984
Transib	5	287
**LTR Retrotransposon**	**14,223**	**4,722,973**
DIRS	2,637	800,713
ROO	2	185
Copia	874	89,153
BEL	3,574	1,341,375
Troyka	85	38,236
Gypsy	11,722	953,269
**Other transposons**	**95,987**	**5,998,951**
Other	95,987	5,998,951
**snRNA**	**20**	**2,169**
snRNA	20	2,169
**Endogenous Retrovirus**	**433**	**28,758**
ERV	433	28,758
**Other**	**21,765**	**5,987,069**
Satellite	292	23,500
Other transposons	21,473	5,963,569
**rRNA**	**957**	**54,960**
SSU-rRNA	84	21,669

rRNA	873	33,291
**Non-LTR Retrotransposon**	**96,263**	**21,946,293**
Proto	10	487
Crack	3,041	707,173
Penelope	30,419	5,797,599
R1	21	839
Nematis	44	10,945
Loa	4	196
RTEX	27	5,254
Rex	2,391	765,528
CRE	2,204	634,127
Jockey	84	15,006
L1	913	280,818
Ingi	6	382
L2	4,781	1,265,995
RTE	2,215	633,292
Nimbus	2	143
7SL	2	629
Outcast	1	94
Perere	4,004	985,433
Tad1	14	771
Tx1	738	327,507
SINE	11,414	1,744,733
R4	106	19,675
Neptune	552	81,827
Daphne	3,346	923,130
Poseidon	6,072	1,425,311
Hero	10	2,268
I	13	719
CR1	23,748	6,296,981
R2	81	19,431
**tRNA**	**28,324**	**3,981,121**
tRNA	28,324	3,981,121
**Unclassified** [c]	**67,853,343**	**67,853,343**
**Low complexity**	**47,807**	**9,252,804**
Other low complexity	6,574	1,609,158
Simple repeat	41,233	7,643,646

[a] Categories of elements are indicated in **bold**.

[b] Subtotals for the respective categories indicated in **bold**.

[c] Previously described, but poorly defined repetitive elements.

Supplemental Figures

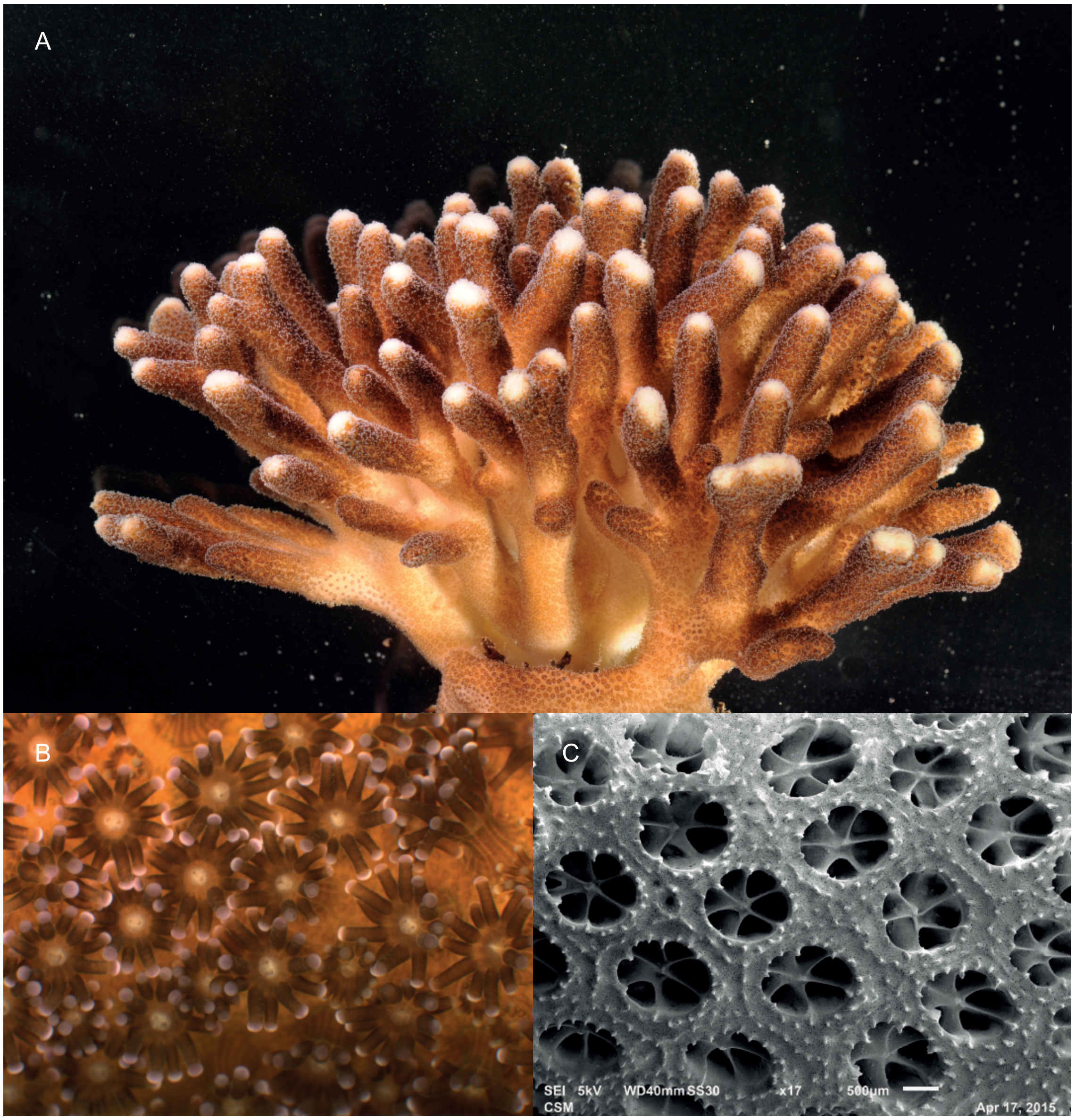

Fig. S1. *Stylophora pistillata* (Esper 1797), clade 4 *sensu* Keshavmurthy *et al.* (2013) (A) Picture of colony in the aquarium at the Centre Scientifique de Monaco (CSM). (B) Magnification of coral polyps. (C) Skeletal corallite structure, scale bar at 500 µm. Pictures kindly provided by E. Tambutté, CSM.

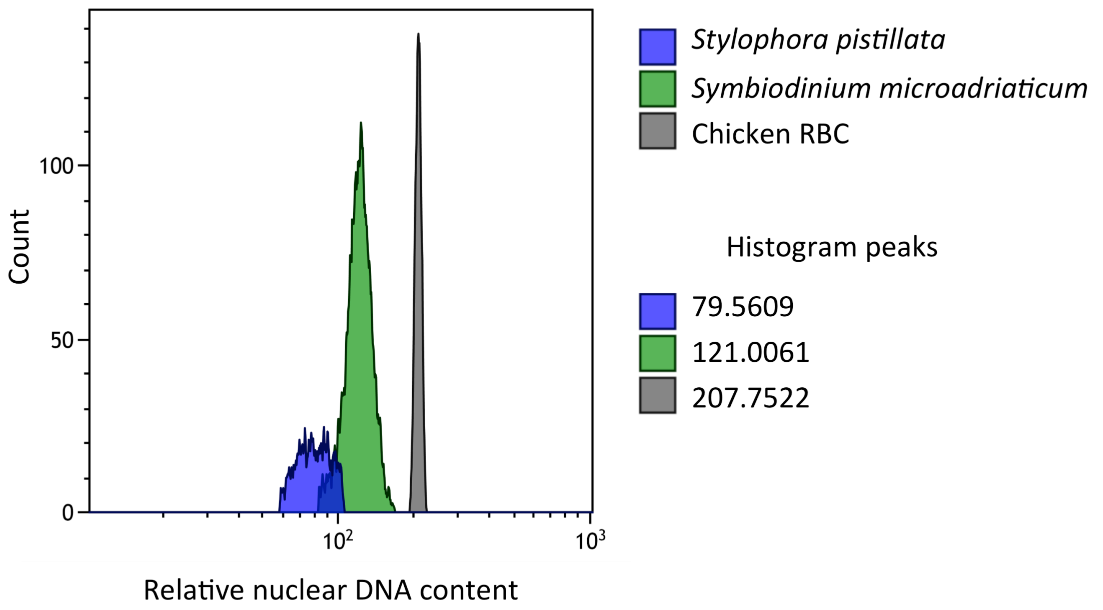

Fig. S2. FACS estimate of the *Stylophora pistillata* genome size. Flow cytometry DNA histograms were obtained with fresh, non-*fixed S. pistillata* cells and mixed with chicken erythrocytes as a reference. The count profile of the Fluorescence-activated cell sorting (FACS) shows three peaks, representing propidium-iodide-stained nuclei of reference chicken erythrocytes (grey), *S. pistillata* (blue), and associated algal symbionts that were not removed from the preparation (*Symbiodinium microadriaticum*, green). Given the known chicken erythrocytes 2C DNA content of 2.33 pg, the haploid DNA content of *S. pistillata* is estimated to be 0.446 pg or ~434 Mbp, while the haploid *S. microadriaticum* genome is estimated to be 1.36 pg or 1,332 Mbp.

Supplemental Datasets

Dataset S1. One-to-one orthologs.

Dataset S2. Many-to-one and many-to-many orthologs.

Dataset S3. Gene Ontology Enrichment of one-to-one orthologs for *S. pistillata* and *A. digitifera*. Only significantly enriched processes ($p < 0.05$) are shown.

Dataset S4. One-to-one orthologs displaying ≥ 50%, between < 50% and ≥ 30%, and those displaying < 30% pairwise sequence similarity.

Dataset S5. Gene Ontology Enrichment (Biological Process) of many-to-one and many-to-many ortholog for S. pistillata and A. digitifera. Only significantly enriched processes ($p < 0.05$) are shown.

Dataset S6. Gene expansion of ortholog groups in *S. pistillata* and *A. digitifera* (n ≥ 3).

Dataset S7. Species-specifc gene expansions of ortholog groups in *S. pistillata* and *A. digitifera* (n ≥ 3).

Dataset S8. Summary table for best hit of each *S. pistillata* gene model vs. SwissProt/TrEMBL/nr. 25,769 genes in total; of which 17,506 have hits to Swiss-Prot, 3,940 to TrEMBL, and 1,466 to nr. 2,857 have no hits to all three databases. E-value cutoff set at 1e-5; Swiss-Prot and TrEMBL hits have at least one GO term assigned to them.

Dataset S9. Summary table for best hit of each A. digitifera gene model vs. SwissProt/TrEMBL/nr. 23,523 genes in total; of which 16,024 have hits to Swiss-Prot, 2,519 to TrEMBL, and 1,428 to nr. 3,551 have no hits to all three databases. E-value cutoff set at 1e-5; Swiss-Prot and TrEMBL hits have at least one GO term assigned to them.

Supplemental References

Keshavmurthy S, Yang S-Y, Alamaru A*, et al.* (2013) DNA barcoding reveals the coral "laboratory-rat", Stylophora pistillata encompasses multiple identities. *Scientific Reports* **3**, 1520.

Shinzato C, Shoguchi E, Kawashima T*, et al.* (2011) Using the Acropora digitifera genome to understand coral responses to environmental change. *Nature* **476**, 320-323.
